# Substitutions at Loop Regions of TMUV E Protein Domain III Differentially Impair Viral Entry and Assembly

**DOI:** 10.3389/fmicb.2021.688172

**Published:** 2021-06-28

**Authors:** Tao Hu, Zhen Wu, Shaoxiong Wu, Mingshu Wang, Renyong Jia, Dekang Zhu, Mafeng Liu, Xinxin Zhao, Qiao Yang, Ying Wu, Shaqiu Zhang, Juan Huang, Sai Mao, Xumin Ou, Qun Gao, Di Sun, Yunya Liu, Ling Zhang, YanLing Yu, Shun Chen, Anchun Cheng

**Affiliations:** ^1^Research Center of Avian Disease, College of Veterinary Medicine, Sichuan Agricultural University, Chengdu, China; ^2^Institute of Preventive Veterinary Medicine, College of Veterinary Medicine, Sichuan Agricultural University, Chengdu, China; ^3^Key Laboratory of Animal Disease and Human Health of Sichuan Province, Chengdu, China

**Keywords:** TMUV, envelope protein, loops of domain III, virus entry, virus assembly

## Abstract

Flavivirus envelope protein (E) plays an important role in cellular infection, especially in virulence and antigenicity. E domain III of Tembusu virus (TMUV) is highly conserved among flaviviruses and contains four loop regions. However, the functions of the loop regions of TMUV E domain III in the viral life cycle have not yet been discovered. In this study, using a reverse genetics system, we performed site-directed mutagenesis on loops I, II, III, and IV of TMUV E domain III. Mutant 6 (S388A.G389A.K390A) showed better proliferation than the wild-type virus, while mutants 1–5 exhibited decreased *in vitro* infectivity, as determined by immunofluorescence assay (IFA). Based on a TMUV replicon system, the mutations exhibited no apparent effect on TMUV RNA replication. Subcellular fractionation assays and packaging system assays indicated that mutations in loops II–IV (T332A, T332S, S365A.S366A.T367A, and S388A.G389A.K390A, respectively) disrupted virion assembly. Moreover, loops I–IV played an important role in virus binding and entry, while mutant 6 (S388A.G389A.K390A) exhibited robust activity in virus entry. Taken together, our findings indicated the critical role of the loop regions in TMUV E domain III in the virus entry and assembly process.

## Introduction

Tembusu virus (TMUV) is a member of the genus *Flavivirus* in the family *Flaviviridae* and was originally isolated from mosquitoes in 1955 (Yu et al., 2018). In 2010, TMUV caused outbreaks in ducks, characterized by a severe drop in egg production and growth retardation (Liang et al., 2019). Similar to other flaviviruses, including West Nile virus (WNV) and Japanese encephalitis virus (JEV), TMUV is an enveloped, positive-sense RNA virus. The genome encodes a single polyprotein, which is cleaved by viral and host proteases into 10 proteins: three structural proteins [core (C), premembrane (prM), and envelope (E) protein] and seven non-structural proteins (NS1, NS2A, NS2B, NS3, NS4A, NS4B, and NS5) (Unni et al., 2011).

Flavivirus infection of host cells is a multistep process (Cruz-Oliveira et al., 2015). The first step of the life cycle is virus binding and entry. Following the entry step, flaviviruses are internalized *via* endocytic pathways at low pH; then, virus nucleocapsids are released into the cytoplasm. The viral genome in the cytoplasm is used for the synthesis of polyproteins, which are processed by viral and host proteins. Genomic RNA is replicated in the replication complex (RC) within a rearranged endoplasmic reticulum (ER)-derived membrane vesicle. When genomic RNA and structural proteins (C,prM, and E) are synthesized, they are assembled in the lumen of the ER and processed into immature virions (Mohd Ropidi et al., 2020). Subsequently, the immature virions are transported *via* a secretory pathway to the trans-Golgi network (TGN) for reprocessing. In this step, the prM protein is processed to mature M by furin. Mature virions are released by exocytosis.

The TMUV E protein, similar to that in other flaviviruses, which are highly structurally conserved, is anchored to the membrane by two transmembrane domains (Kostyuchenko et al., 2016). The extracellular domain of the E protein is composed of three domains and linked to transmembrane domains by a stem region. Domain I participates in E protein conformational changes and stability. Domain II contributes to virus-mediated membrane fusion and contains cross-reactive epitopes and neutralizing antibody epitopes. Domain III is an immunoglobulin-like structure that is thought to interact with cell receptors, and it contains four loop regions (Zhang et al., 2017). The stem and transmembrane regions are related to conformational changes in the E protein and participate in the process of membrane fusion (Allison et al., 1999; Fritz et al., 2011).

Domain III of flavivirus is involved in the receptor-mediated entry process and has virulence-determining sites and antibody recognition epitopes. Recombinant domain III proteins lead to inhibited virus entry (Fan et al., 2013). The Arg380 residue of the yellow fever virus (YFV) E protein has been proven to be a key site for the binding of the virus to cell glycosaminoglycans (GAGs), which are among the important attachment factors of flaviviruses (Lee and Lobigs, 2008). Residues K291 and K295 in domain III of dengue virus (DENV) are involved in the E–GAG interaction (Watterson et al., 2012). Substitutions of amino acids 363–367 can prevent virus release but can enhance entry activity (Liu et al., 2015). The E-367 mutation (T→K) in a TMUV live attenuated vaccine candidate has been previously shown to affect infection *in vitro* and *in vivo*, and a substitution with a basic amino acid can enhance its binding affinity for GAGs (Sun et al., 2020). The E-388-390 motif [Arg-Gly-Asp (RGD)] exists in many flaviviruses (such as JEV) and is important for virus binding with host cell integrin (van der Most et al., 1999). The E-390 mutation in Murray Valley encephalitis virus (MVE) can attenuate viruses by altering entry kinetics (Lee and Lobigs, 2000). A recent study found that substitution at TMUV E-304 can alter its virulence, and the substitution of basic residues can markedly increase the affinity of the virus for GAGs (Yang et al., 2021). A YFV variant that escapes neutralization by a monoclonal antibody (mAb) exhibited a conformational change in the E protein, and the E-305 residue was identified to be one of the epitopes bound by the mAb and one of the determinants of YFV pathogenesis *in vivo* (Ryman et al., 1998). A panel of WNV mutants containing all possible amino acid substitutions at E-332 revealed that the change in E-332 had a great influence on the antigenicity of the virus (Plante et al., 2016). The amino acid change in the linker between domain I and domain III in DENV impaired virus assembly (de Wispelaere and Yang, 2012). However, the function of TMUV in general and that of flavivirus domain III loop regions in particular in the viral life cycle are not fully understood.

To probe the function of the loop regions of the TMUV E protein in the viral life cycle, we chose residues located in loop regions (loop I, M304.C305.S306 and V312; loop II, T332; loop III, S365.S366.T367; and loop IV, S388.G389.K390) and replaced conserved amino acids. In this study, we then used a reverse genetic system to construct and rescue the mutant viruses. We used immunofluorescence assay (IFA) and the number of infectious particles to analyze rTMUV proliferation. A subcellular fractionation and packaging system was used for analyzing the virus assembly step. The entry step was studied by binding and entry assays. Here, we show that these mutations had an effect on viral proliferation. Further characterization of the phenotype of these mutants revealed that the loop regions of TMUV E protein domain III differentially impaired viral entry and assembly.

## Materials and Methods

### Cells and Antibodies

Baby hamster kidney cells (BHK-21) were obtained from the American Type Culture Collection (ATCC) and were grown in Dulbecco’s modified Eagle’s medium (DMEM) (Gibco Life Technologies, shanghai, China) supplemented with 10% fetal bovine serum (FBS; Gibco Life Technologies, shanghai, China) in 5% CO_2_ at 37°C.

Mouse anti-TMUV serum was produced by our laboratory previously (Chen et al., 2018a). Antibody against TMUV E mAb was produced by our laboratory. Rabbit anti-Calnexin pAb was obtained from Abcam (ab22595). Mouse anti-β-actin mAb was obtained from Transgen. Goat anti-mouse IgG [horseradish peroxidase (HRP), fluorescein isothiocyanate (FITC)] used as the secondary antibody was obtained from Thermo Fisher Scientific (USA).

### Infectious Clones, Replicons, and C-prM-E Constructs

The infectious cDNA clone of TMUV (CQW1 strain) used in this study, pACYC-CQW1, was constructed by our laboratory (Chen et al., 2018a). A subclone, pACYC-CQW1-P1, containing the *Spe*I-*Xho*I fragment of pACYC-CQW1 (nucleotides 1–2671 of the viral genome), was used to engineer mutations using a Fast Mutagenesis System (Transgen), and the mutated DNA fragments were cloned back into pACYC-CQW1 with *Spe*I and *Xho*I. The primers used for mutagenesis are listed in Table 1.

**TABLE 1:** PCR primers used in this research.

Primer^a^	Sequence (5′–3′)	Purpose
Mutant 1-F	AATGACCTACCCGGCGGCTGCCAATACATTT	Introduced mutation
Mutant 1-R	GCAGCCGCCGGGTAGGTCATTCCTTTC	
Mutant 2-F	AATACATTTTCCCTAGCGAAGAATCCTA	
Mutant 2-R	GCTAGGGAAAATGTATTGCTACACATC	
Mutant 3-F	AATTGTCTTATGCAGGTGCCGATGGGCC	
Mutant 3-R	CACCTGCATAAGACAATTCCACCACGAC	
Mutant 4-F	AATTGTCTTATGCAGGTTCCGATGGGCC	
Mutant 4-R	AACCTGCATAAGACAATTCCACCACGAC	
Mutant 5-F	ATACGTGTCGACTGCCGCCGCGGGTGCCAA	
Mutant 5-R	CGGCGGCAGTCGACACGTATGGGTTGACTGTTA	
Mutant 6-F	TTATTTTAGTAGGAGCTGCAGCAGGACAGATTAG	
Mutant 6-R	GCTGCAGCTCCTACTAAAATAAATGAATCCC	
TMUV-E F	AATGGCTGTGGCTTGTTTGG	qRT-PCR
TMUV-E R	GGGCGTTATCACGAATCTA	
duck β-actin-F	GATCACAGCCCTGGCACC	
duck β-actin-R	CGGATTCATCATACTCCTGCTT	
Clone-*Spe*I-F	ACACTCCGCTAGCATACTAGTTAATACGACTC ACTATAGGGAGAAGTTCA	One-step cloning
Clone-*Xho*I-R	TTACCCACATATTGTGCTCGAGC CTGCTGACTGATC	

The plasmid pCDNA3.1-C_16_prME and replicon-Nluc used for packaging system was constructed previously (He et al., 2019). The pCDNA3.1-C_16_prME plasmid was mutagenized using the primers listed in Table 1.

### RNA Transcription *in vitro*, Transfection, and Indirect Immunofluorescence Assay

Plasmids were extracted using Hipure plasmid mini kit (Magen) and linearized with the restriction enzyme *Sma*I. Linearized plasmids were transcribed to mRNA using mMESSAGE mMACHINE^TM^ T7 KIT (Thermo Fisher Scientific) according to the manufacturer’s instructions.

IFA was performed as previously reported (Chen et al., 2018a). Briefly, BHK-21 cells were seeded on coverslips that were placed in 12-well plates. The cells were transfected with RNA using LIPOFECTAMINE MESSENGERMAX^TM^ (Invitrogen). At 72 h post-transfection, cells were fixed in 4% paraformaldehyde for 1 h and permeabilized with 0.25% Triton X-100 for 1 h in 4°C. And the cells were blocked with 5% bovine serum albumin (BSA) in Phosphate Buffer Solution (PBS) for 1 h after three washes with PBS, then incubated with mouse anti-TMUV serum. After incubation, the cells were washed with PBS again, incubated with secondary antibody and 4’,6-diamidino-2-phenylindole (DAPI, Coolaber), and visualized using a microscope.

To verify the effect of trypsin on the removal of attachment viruses, TMUV and BHK-21 cells were incubated at 37°C for 1 h, and then washed three times with PBS or 0.025% trypsin-PBS, respectively. The cells were blocked with 5% BSA in PBS for 1 h and then incubated with mouse anti-TMUV serum, washed, incubated with secondary Abs and DAPI (Coolaber), washed, and visualized using a microscope.

### RNA Extraction and Quantitative Reverse Transcription PCR

Viral RNAs in cultural supernatant were extracted using TIANamp Virus DNA/RNA Kit (TIANGEN), and intracellular total RNAs were extracted using RNAiso Plus reagent (TARAKA). The RNAs were transcribed using HiScript^®^ III RT SuperMix (Vazyme). The number of genome-containing particles (G) was determined by absolute quantitative PCR according to the qPCR procedure previously established in our laboratory (Chen et al., 2018b).

### Virus Titration

Virus infectivity was determined by the median tissue culture infectious dose 50 (TCID_50_) method on BHK-21 cells. Viral sample was 10-fold serially diluted in DMEM containing 1% penicillin/streptomycin (Solarbio), and 100-μl dilutions were added to 96-well plates seeded with BHK-21 cells. Viral titers were calculated according to the Reed and Muench method.

### Sodium Dodecyl Sulfate–Polyacrylamide Gel Electrophoresis and Western Blot Analysis

Proteins were harvested with RIPA buffer (Thermo Fisher Scientific) and denatured in 6 × protein loading buffer (TransGen) and incubated for 10 min at 95°C. Equal samples were separated by sodium dodecyl sulfate–polyacrylamide gel electrophoresis (SDS-PAGE) (10% polyacrylamide) and transferred onto polyvinylidene fluoride (PVDF) membranes by using Mini-PROTEAN Tetra System (Bio-Rad). Membranes were washed three times by TBST buffer (10 mM Tris-HCl, pH 7.5, 150 mM NaCl, and 0.5% Tween 20) and blocked with 5% non-fat milk in TBST and incubated with the primary antibodies (Calnexin 1:2,000; E 1:1,000) by overnight incubation at 4°C. After washing with TBST three times, membranes were incubated with the secondary HRP-conjugated antibodies and visualized using enhanced chemiluminescence (ECL) system (Bio-Rad).

### Tembusu Virus Packaging System

BHK-21 cells were plated in 24-well plates and co-transfected with replicon-Nluc and pCDNA3.1-C_16_prME plasmids using Lipofectamine 3000 (Thermo Fisher Scientific) according to the manufacturer’s instructions. The cell lysates were collected at 4 and 9 h post-transfection. For virus assembly and release assay, BHK-21 cells were transfected with replicon-Nluc; after 36 h of transfection, the packaging plasmids were transfected. Cell lysates were collected at 24 h post-transfection. The cell samples were lysed using Glo lysis buffer (Promega) at room temperature, and Nluc activity was detected using Nano-Glo Luciferase Assay system and GloMax Navigator System (Promega).

### Subcellular Fractionation

Subcellular fractionation was performed as described previously (Liu et al., 2015). BHK-21 cells transfected with wild-type (WT) or mutant transcribed RNA were trypsinized and washed in PBS three times. The cell pellets were resuspended in hypo-osmotic buffer (10 mM HEPES-NaOH, pH = 7.8). The cells were allowed to swell on ice for 10 min, then centrifuged at 800 g for 2 min at room temperature. The samples were returned to iso-osmoticity by removal of 650 μl of the supernatant and the addition of 350 μl of hyperosmotic buffer (0.6 M source, 10 mM HEPES-NaOH, pH = 7.8). The cells were disrupted by passage 50 times through a 25-G needle, and nuclei were removed by centrifugation for 30 min at 13,000 g at 4°C. The postnuclear fraction was mixed with 700 μl of 60% iodixanol to generate a solution containing 30% iodixanol. Solutions containing 10 and 20% iodixanol were generated by mixing with hypo-osmotic buffer. A total of 4.2 ml of these three solutions were layered in centrifuge tubes, which were centrifuged at 50,000 rpm in an S52-ST rotor (Thermo Fisher Scientific) for 3 h at 4°C. Ten fractions of total 420 μl were collected from top to bottom, and 200 μl was extracted RNA for qPCR, 100 μl for Western blot analysis. The remaining 100 μl of each fraction was titrated by TCID_50_.

### Virus Attachment and Entry Assay

For entry assay, BHK-21 cell monolayers in 12-well plates were used. Rescued virus samples (equal amounts of GCPs) were added to each well at room temperature. At 1 h after virus addition at 37°C, cells were washed three times with 0.025% trypsin-PBS. And the trypsin was aspired and replaced with DMEM containing 2% FBS. The trypsinization can inactivate virus that has not been endocytosed on the cell surface. For additional 1 h cell culture, total RNA was extracted from cells using TRIzol reagent (TAKARA). qPCR was performed to quantify viral RNA.

For the virus binding assay, BHK-21 cells were cooled to 4°C for 1 h, then the medium was replaced with equal GCPs WT or mutant virus for 1 h at 4°C. Unbound virus was removed by washing with precooled PBS three times. Total RNA was extracted from cells to quantify viral RNA.

## Results

### Selection of Amino Acids in the Envelope Protein Is Potentially Involved in the Virus Life Cycle

**FIGURE 1:**
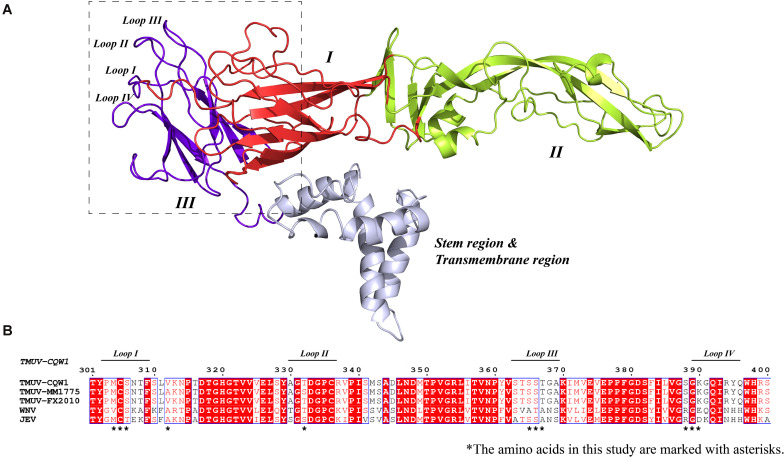
**(A)** Side view of structural conformation of E protein monomer, the homology model building of Tembusu virus (TMUV) E protein based on the resolved cryo-EM structure of the Zika virus (ZIKV) E protein (PDB ID: 5IZ7) using SWISS-MODEL. The domain I, domain II, domain III, and stem region and transmembrane region are shown in red, lemon yellow, purple-blue, and blue white. **(B)** Alignment of E protein sequences from TMUV (CQW1: AIU44176.1, FX2010: AWV66902.1, MM1775: AWV66903.1), West Nile virus (WNV) (NP_776014.1), and Japanese encephalitis virus (JEV) (BAD81042.1) using MEGA7, and ESPript was used for graphical enhancements. The loop regions in A are marked with a horizontal line. The selected amino acids in this study are marked with asterisks.

In order to better understand the structure of TMUV E protein, we used the known Zika virus (ZIKV) E protein structure as a template for homology modeling and obtained the predicted structure of TMUV E protein (Kostyuchenko et al., 2016; Figure 1A). In flavivirus E protein, domain III was considered the dominating part of cell–virus interplay, and some residues in domain III related to virulence (Chin et al., 2007; Nickells et al., 2008; Zhang et al., 2017). Sequence alignment of E protein of various flaviviruses revealed that the amino acid sequence of domain III is relatively conserved; however, in the four loop regions, the sequence is different (Figure 1B). Studies have shown that these special sites are related to the recognition of neutralizing antibodies and host receptor molecules (Chiou et al., 2012; Watterson et al., 2012). The E-306, 332, 366 can be recognized by antibodies that could recognize mature virus, indicating that these residues are crucial participants in the formation of mature virus conformation (Lee et al., 2130). The conserved C305 was identified as participating in the formation of disulfide bonds, which are essential for stabilizing the structure of E protein; however, it is still unclear which viral life cycle it is involved in Du et al. (2015). In view of this, we selected the amino acid located in the central part of the domain III loop regions for subsequent research [mutant 1, M304.C305.S306 (Loop I); mutant 3, T332 (loop II); mutant 5, S365.S366.T367 (loop III); mutant 6, S388.G389.K390 (loop IV)] (Table 2). In addition, as a flavivirus, TMUV was thought to be transmitted by mosquitos. A previous study showed that TMUV isolates derived from poultry were more aggressive and more likely to infect mammalian cells than isolates derived from mosquitoes (Yan et al., 2018). The same study also revealed that two viruses have different tissue tropisms, and the E protein is the determinant of tissue tropism. The MM1775 chimeric virus with duck-derived TMUV E protein acquired the ability to be transmitted by contact in duck flocks (Yan et al., 2018). Because the two viruses in the previous study had different infection characteristics in ducks and since E protein domain III is a possible determinant of virulence, we compared the sequences of E protein domain III of two strains. Considering the sequence alignment of the TMUV CQW1 strain and MM1775 strain, we introduced two additional mutations [mutant 2, V312 (which is different from residue 312 in the MM1775 strain); mutant 4, T332 (which is different from the 332 residue in the MM1775 strain)] (Table 2).

**TABLE 2:** Mutations introduced into the Tembusu virus (TMUV) E protein.

Mutant	Position or regions	Amino acids substitutions
Mutant 1	M304.C305.S306 (*Loop I*)	MCS→AAA
Mutant 2	V312 (Different from MM1775 strain)	V→A
Mutant 3	T332 (*Loop II*, different from MM1775 strain)	T→A
Mutant 4	T332 (*Loop II*, different from MM1775 strain)	T→S
Mutant 5	S365.S366.T367 (*Loop III*)	SST→AAA
Mutant 6	S388.G389.K390 (*Loop IV*)	SGK→AAA

### Amino Acid Substitutions Affect the Proliferation of Tembusu Virus

**FIGURE 2:**
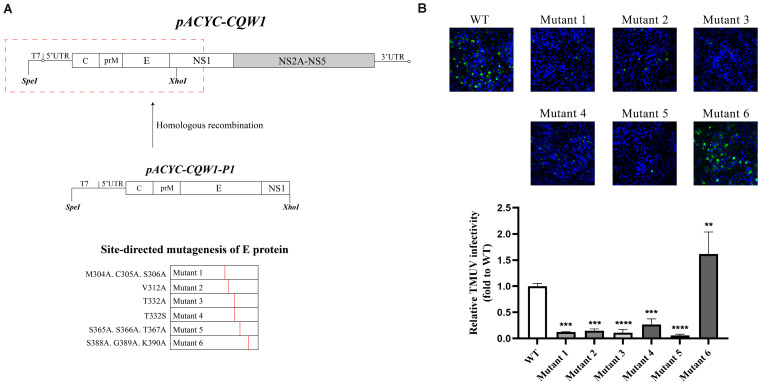
The engineered mutations introduced in E protein affected the proliferation of the Tembusu virus (TMUV). **(A)** Schematic representation of infectious clone construction. The subclone pACYC-TMUV-P1, which includes the TMUV C, prM, E, and partial NS1 sequence, serves as a template for introducing mutations. Restriction enzyme sites *Spe*I and *Xho*I are used for fragment cloning. The fragments and pACYC-TMUV are connected by homologous recombination. **(B)** BHK-21 cells were transfected with *in vitro*-transcribed genomic RNAs and then analyzed by immunofluorescence straining at 72 h post-transfection, and mouse anti-TMUV serum polyclonal antibody was used as primary antibody. Green, TMUV; blue, nucleus. Results from one representative experiment out of three independent experiments are shown (upper). Quantification of virus infection rates by ImageJ (lower). Means and standard errors are from three microscopic field of views. Asterisks denote a statistically significant reduction in viral proliferation compared to that of the wild type (WT) (*p ≤ 0.05; **p ≤ 0.005; ***p ≤ 0.001; ****p ≤ 0.0001).

To investigate the impact of selected amino acid changes on the viral life cycle, the codons for the residues in the TMUV full-length infectious clone pACYC-CQW1 were mutated. To avoid redundant mutations in the process of introducing engineering mutations, we used a subclone, pACYC-CQW1-P1, containing the CprME protein sequence with *Spe*I and *Xho*I restriction sites. We performed site-directed mutagenesis on pACYC-CQW1-P1 and then cloned the P1 fragment onto pACYC-CQW1 (Figure 2A). Finally, we successfully generated six mutant viruses (mutants 1–6; Figure 2A).

**FIGURE 3:**
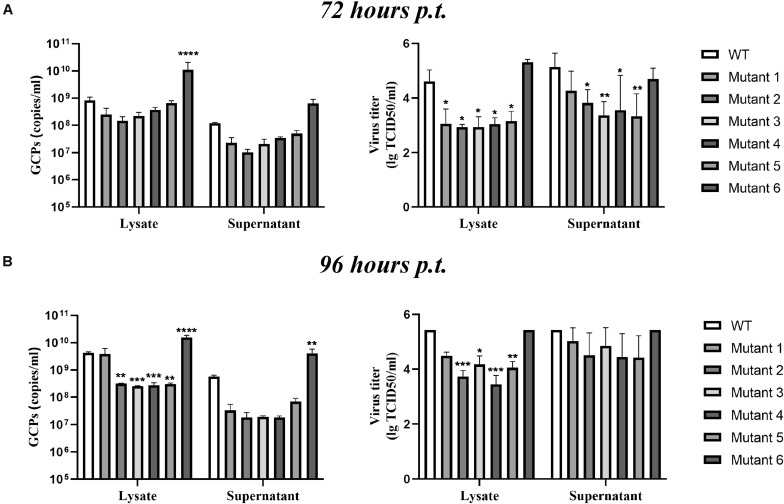
Infectious virus production in BHK-21 cells. The viral titer and genome-containing particle (GCP) level of the cell lysate and supernatants collected at 72 h post-transfection (p.t.) **(A)** and 96 h p.t. **(B)** were quantified by TCID_50_ and qPCR. Means and standard errors are from three independent experiments. *p ≤ 0.05; **p ≤ 0.005; ***p ≤ 0.001; ****p ≤ 0.0001.

After completing the correct introduction of mutations, the plasmids of pACYC-CQW1 were linearized and then transcribed into RNA *in vitro*. Equal amounts of *in vitro*-transcribed RNA were transfected into BHK-21 cells, and IFA was performed 72 h post-transfection. The IFA-positive cells in the WT and mutant groups were clearly different. Mutants 1–5 showed a dramatic reduction in viral proliferation, and the viral infectivity of mutant 6 was obviously increased or equal to that of the WT virus (Figure 2B). To better evaluate the proliferation and infectivity of the mutant viruses in BHK-21 cells, we quantified the infectious virus particles 72 h post-transfection, and the lysates and culture supernatants from transfected cells were quantified by qRT-PCR and the TCID_50_ was determined. Consistent with the IFA results, the groups of low IFA-positive cells had significantly low GCP numbers and viral titers in both the cell lysate and supernatant (Figure 3A). Correspondingly, compared with the WT virus, the GCP number of mutant 6 was 13-fold in lysate and 5.46-fold in culture medium, while the titer was comparable to that of WT TMUV. Since we observed a remarkable change in mutant virus proliferation 72 h post-transfection, we tried to confirm whether this decreasing or increasing trend changes over time. Therefore, we further tested the level of the viral genome and infectivity 96 h post-transfection. Although the viral genome and titer of the mutants increased with infection time, their levels were still lower than those of the WT virus. Specifically, mutant 6 showed better proliferation than WT TMUV (Figure 3B). We sequenced and analyzed the mutant viruses 5 days post-transfection and found no additional mutations in the E gene. Overall, the results indicated that most mutations impaired virus production.

### The Change in Virus Proliferation Is Not Due to Changes in Viral Genome Replication

**FIGURE 4:**
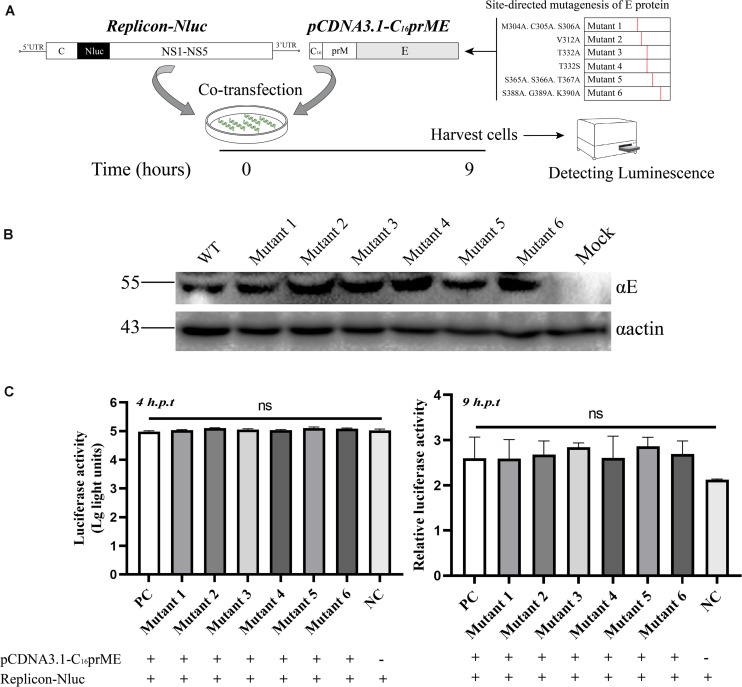
The effects of the structure protein with E mutations on viral RNA replication using Tembusu virus (TMUV) replicon system. **(A)** Schematic diagram for the replication assay. **(B)** BHK-21 cells were transfected with wild-type (WT) or mutant packaging plasmid. Cells were harvested for Western blot (WB) at 18 h post-transfection (p.t.) with the indicated Abs. **(C)** Luciferase activities of BHK-21 co-transfected replicon-Nluc and pCDNA3.1-C_16_prME on 4 h p.t. (left panel). The relative luciferase activity is represented by the Nluc level at 9 h p.t. relative to 4 h p.t. The Nluc levels of all mutants are shown as relative ratios to wild type (WT) (right panel). PC: WT pCDNA3.1-C_16_prME, NC: replicon-Nluc only. Means and standard errors are from three independent experiments. ns, no statistically significant difference in viral genome replication.

A complete life cycle of a flavivirus involves virion attachment, entry, uncoating, genome replication, and virus particle assembly and release. To rule out E protein mutation disruption of viral genome replication, we analyzed genome replication using the TMUV packaging system as previously reported (He et al., 2019). The flavivirus packaging system has been shown to be a model system to study viral entry, assembly, and release (Gehrke et al., 2003; Lin et al., 2011). It consists of a packaging plasmid containing structural proteins and a replicon that provides the indispensable units for genome replication. To exclude the effect of E protein mutations on viral genome replication, site-directed mutagenesis was carried out in the packaging plasmid, pCDNA3.1-C_16_prME (Figure 4A). We verified the protein expression of the mutant packaging plasmid, and no E protein expression was found to be affected (Figure 4B). The packaging plasmid and replicon were cotransfected into BHK-21 cells, and then, the Nluc activity level was detected because it represents the replication level of the replicon (Figure 4A). As the time after transfection increases, the packaging plasmids wrap the replicon into single round infectious particles (SRIPs). We chose a relatively early time point that represents the low-generation replication of replicons to prevent the replicon from being released outside the cell in the form of SRIPs (Figure 4C). The Nluc activity levels were determined 4 h post-transfection, representing transfection efficiency (Figure 4C, left). Then, the Nluc activity levels were measured 9 h post-transfection, and the relative luciferase activity represented viral genome replication (Figure 4C, right). The Nluc ratios of the six mutants were comparable to the Nluc ratio of the WT. These data suggest that the mutations did not interfere with the replication of the viral genome.

### Tembusu Virus Domain III Mutations Impair Virus Assembly

**FIGURE 5:**
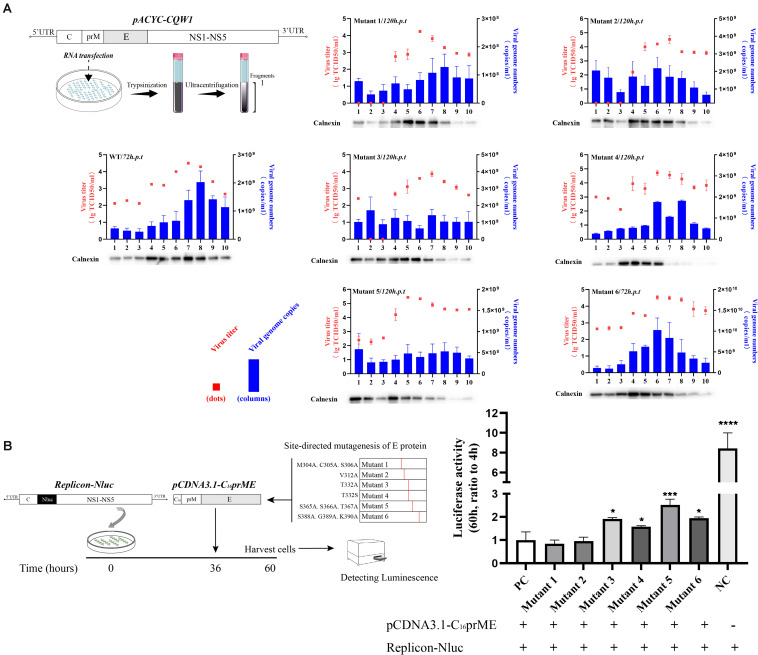
Subcellular fractionation of *in vitro*-transcribed genomic RNA-transfected BHK-21 cells and packaging system assay. **(A)** Schematic diagram for the subcellular fractionation assay (left panel). The cellular contents were analyzed by fractionation on an iodixanol gradient. Ten fractions were collected from the top of the gradient, and the individual fractions were titrated by TCID_50_ (red dots). The blue columns represent the distribution of infectious virus in the fractions collected from cells transfected with Tembusu virus (TMUV) RNA. Each fraction was also chloroform/methanol precipitated and analyzed by Western blot (WB) for the presence of calnexin. **(B)** Schematic diagram for the packaging system assay (left panel). The relative luciferase activity is represented by the Nluc level at 60 h post-transfection relative to 4 h post-transfection. The Nluc levels of all mutants are shown as relative ratios to wild type (WT). PC: WT pCDNA3.1-C_16_prME, NC: replicon-Nluc only (right panel). Means and standard errors are from three independent experiments. Asterisks denote a statistically significant difference in viral assembly compared to that of the WT. *p ≤ 0.05; **p ≤ 0.005; ***p ≤ 0.001; ****p ≤ 0.0001.

To examine the effect of these mutations on the formation of infectious particles, we fractionated transfected BHK-21 cell lysates using gradient centrifugation (Figure 5A, left). Seventy-two hours (WT and mutant 6) or 120 h (mutants 1–5) post-transfection, fractions were collected from top to bottom. We analyzed the number of infectious particles and the viral genome number contained in each fraction. In addition, we analyzed the calnexin level in each fraction because it represents the assembly sites of virus particles on the ER. For flaviviruses, the majority of the calnexin generally accumulates in fractions 4 through 8, and infectious virus particles and virus genomes have been previously detected in these fractions (de Wispelaere and Yang, 2012; Liu et al., 2015). Our results were consistent with these studies (Figure 5A, right). The consistency of the three indicators reflects the correct assembly of virus particles on the ER. For mutant 4, although there was a peak in the number of genomes in fraction 8 and the distribution of infectious virus particles was similar to that of WT, the ER marker protein showed a component shift (Figure 5A, right). We presumed that the packaging sites of mutant 4 on the ER changed, which negatively affected assembly efficiency. For mutant 3, the assembly indicators of virions changed in all three respects. The significant distribution of ER markers shifted slightly among fractions, and the level of the viral genome in each fragment not only declined but also appeared to be more evenly distributed than that of the WT virus (Figure 5A, right). We tried to clarify the distribution of the E protein of each component to further analyze the packaging of the virions. However, due to the poor recognition of TMUV E antiserum, the results obtained were ambiguous. According to a previous study, this shift may be attributed to the inability of particles to be released into the secretory pathway (Yu et al., 2008). In the case of mutant 5, the virus titer was redistributed among fractions, while the virus genome was absent in all fractions (Figure 5A, right). For mutant 6, the three indicators were shifted to the fraction with the least density, and the changes were consistent (Figure 5A, right). We concluded that T332A, T332S, S365A.S366A.T367A, and S388A.G389A.K390A severely disrupted the assembly of virus particles, while M304A.C305A.S306A and V312A did not affect the assembly step.

To confirm the effect of E protein mutation on virus particle assembly, we applied a packaging system. After SRIPs were produced by the packaging system, they reenter cells and generate redundant fluorescent signals that interfere with the detection of first-round replication signals. In this experiment, we first transfected the replicon into cells for 36 h and then transfected the packaging plasmid for 24 h. We chose to detect the intracellular luciferase level 24 h after transfection of the packaging plasmids (Figure 5B, left) because the replicon expresses a high level of luciferase 36 h post-transfection, which can dilute the redundant luciferase signal caused by reentry of the SRIPs. In the empty control (NC) group, the level of relative Nluc activity was 8.4-fold that of the experimental control group (pCDNA3.1-C_16_prME and replicon cotransfection). This result indicates that the packaging system was efficient and that the reinfection of SRIPs has little effect on the measures of intracellular Nluc activity. The luciferase signals of mutants 3, 4, 5, and 6 were from 1.5- to 2-fold higher than that of the positive control (PC) (Figure 5B, right). This is consistent with the conclusions of subcellular fractionation experiments. For mutants 1 and 2, the Nluc activity level was similar to that of the PC (Figure 5B, right). Collectively, these data suggest that T332A, T332S, S365A.S366A.T367, and S388A.G389A.K390A caused a major defect in the assembly of viral particles.

### Tembusu Virus Domain III Mutations Impaired Virus Binding and Entry

**FIGURE 6:**
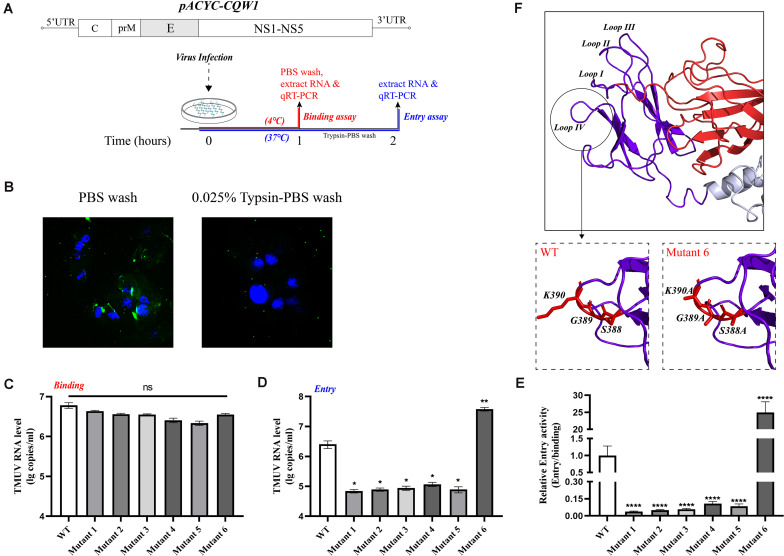
Binding and entry activity of mutant viruses. BHK-21 cells were incubated with equal amounts of wild-type (WT) or mutant viruses. **(A)** Schematic diagram for the binding and entry assay. **(B)** Here, 0.025% trypsin-Phosphate Buffer Solution (PBS) can effectively clear the virus particles on the cell surface. Green, Tembusu virus (TMUV); blue, nucleus. **(C)** For attachment assay, viruses and cells were incubated for 1 h at 4°C, and the unbound viruses were removed by washing with PBS three times. The binding viral RNA of each mutant and WT was analyzed by qPCR. **(D)** For entry assay, virus and cells were incubated for 1 h at 37°C, and the cells were washed with 0.025% trypsin-PBS, then the cells were incubated at 37°C for an additional hour for the full entry of viral particles. The viral RNA of each mutant and WT was analyzed by qPCR. **(E)** The relative entry activity of each mutant was measured by the ratio of entry viral RNA to bound viral RNA. **(F)** Upper panel: Enlargement of the domain III, the loop regions are marked *Loops I–IV*; lower panels: the S388A.G389A.K390A mutation caused the predicted lateral ridge conformation to become smoother. Means and standard errors are from three independent experiments. Asterisks denote a statistically significant difference compared to the WT. *p ≤ 0.05; **p ≤ 0.005; ***p ≤ 0.001; ****p ≤ 0.0001.

Since a major function of the E protein is to mediate flavivirus entry, we questioned whether these engineered mutations of the E protein would have a great impact on the production of infectious particles and whether this effect is related to the entry process of the virus. Moreover, we wanted to clarify the mechanism by which the proliferation of mutant 6 was increased. To examine the effects of these mutations on virus attachment and entry processes, we exploited relative qPCR to measure the viral genomic RNA associated with the cells. First, BHK-21 cells were transfected with *in vitro*-transcribed RNA, and the supernatant was harvested when the cells exhibited 75% cytopathic effect (CPE). Then, the BHK-21 cells were incubated with equal amounts of WT or rescued mutant viruses at levels normalized by qPCR. For the virus binding assay, virus was incubated on cell monolayers at 4°C for 1 h, and unbound virus was removed by washing with precooled PBS. We measured the bound viral genome RNA (Figure 6A). As shown in Figure 6C, mutants 4 and 5 exhibited approximately 50% of the binding ability of the WT virus, and mutants 1, 2, 3, and 6 had a 30% reduction in binding activity; however, there was no statistically significant difference between the mutants and the WT virus. To detect viral entry, we initialized viral internalization into cells for 1 h at 37°C and removed the virus from the cell surface by washing with a 0.025% trypsin-PBS solution. As shown in Figure 6B, 0.025% trypsin-PBS effectively cleared the virus particles from the cell surface. The cells were cultured at 37°C for an additional hour to allow the virus particles to enter the cells completely (Figure 6A). Then, intracellular RNA was extracted and used to detect the level of the virus genome. As expected, the mutations at M304.C305.S306, V312, T332, and S365.S366.T367 clearly disrupted the entry process of rTMUV, and the entry capacity was attenuated by 60% or more, which shows a cumulative result with attachment process (Figure 6E). However, the entry ability of mutant 6 was increased by approximately 14.8-fold compared to that of the WT virus (Figure 6D). Comparing the binding activities of mutant 6 and the WT virus, we found that the effective entry activity of mutant 6 was much higher (Figure 6E). Therefore, we suggest that the increased proliferation ability of mutant 6 was due to its highly effective entry. Taken together, these data demonstrate that M304A.C305A.S306A, V312A, T332A, T332S, and S365A.S366A.T367A disrupt virus binding and entry, especially in terms of relative entry activity, S388A.G389A.K390A promotes virus entry but only slightly interferes with virus binding.

## Discussion

The E protein of flavivirus is critical for virus binding, entry, membrane fusion, and virion formation and is crucial for viral virulence, stability, and tissue tropism (Zhang et al., 2017). With artificial modifications or by natural selection, certain amino acids may be mutated, and these mutations can cause the phenotype of the virus to change (Ramond et al., 2019). This variation may manifest as a change in the infectivity of the virus *in vitro*, virulence *in vivo*, or the recognition of neutralizing antibodies (NAbs) (Ryman et al., 1998). In domain III of flavivirus E protein, a number of decisive sites involved in NAb recognition or virus infectivity have been previously identified (Mandl et al., 2000; Matsui et al., 2010). An increasing number of studies have also shown that virulence attenuation sites and NAb recognition sites are potentially related to the entry process of flaviviruses, even more life cycle steps. We focused on the amino acids in the loop regions because these regions potentially participate in the viral life cycle, especially the entry process. This study predicted the loop region of TMUV E protein domain III based on the crystal structure of the ZIKV E protein and analyzed the effect of the loop region elements on the viral life cycle. The results showed that domain III plays an important role in both viral entry and assembly. Amino acid substitutions in loops I–IV (M304A.C305A.S306A, V312A, T332A, T332S, and S365A.S366A.T367A) attenuate rTMUV replication in BHK-21 cells *in vitro* because the assembly (T332A, T332S, and S365A.S366A.T367A) or entry process (M304A.C305A.S306A, V312A, T332A, T332S, and S365A.S366A.T367A) is disrupted. Surprisingly, alanine replacement at 388–390 enhanced TMUV in BHK-21 cells by facilitating the entry process. These results are consistent with previous reports showing that mutations within domain III of flavivirus exhibited reduced virulence, and partial mutations caused changes in the entry step of the viral life cycle (Lee and Lobigs, 2000). This study also revealed that the loop regions of the TMUV E protein participate in virus assembly. Moreover, C305 participates in the formation of disulfide bonds with the stable E protein; therefore, the disruption of C305 by alanine mutation has been previously shown to be an important reason for the attenuation (Du et al., 2015; Kostyuchenko et al., 2016).

In this study, we found that amino acid substitutions in loop regions of the TMUV E protein affect viral proliferation but not genome replication. In the process of virus particle formation, structural proteins (CprME) and the viral genome are distributed in the assembly sites of the ER. The correct distribution of the two viral components is critical to this step. In the work by de Wispelaere and Yang (2012), the peak of the WT infectious particles accumulated in fractions 4 through 6; correspondingly, the highest peak of the ER marker and prME protein appeared in fractions 4–6. In the work by Liu et al. (2015), although the peak fraction of JEV infectious particles was slightly different from that of DENV2 (there was only one peak of infectious particles in JEV), the overall distribution trend was consistent. We found that for the titer, genome level, and calnexin level, a deviation in the distribution of any two of these measures significantly affected the packaging of the virion. Therefore, we chose to detect virus titer, genome level, and ER marker in different fractions obtained by subcellular fractionation to determine the assembly of virus particles. Both mutants 1 and 2 underwent changes in assembly speed but not capability. In addition, in the packaging system assay, their Nluc activity levels were similar to those of the WT virus, which indicates that the packaging system can more intuitively indicate the assembly of the mutant virus. Mutants 3, 4, and 5 also showed disrupted virus assembly, and the packaging system amplified these defects because the genome was more evenly distributed (in mutants 3 and 5) or the packaging site was severely shifted (in mutants 4 and 5) (Table 3). Unexpectedly, although we found that mutant 6 showed enhanced infectivity, the mutations partially interfered with virus assembly or release. We think that this is why the titer of the mutant 6 variant at 72 h post-transfection in the supernatant was lower than that of the WT.

**TABLE 3:** Summary of the effects of mutations on virus assembly, RNA replication, binding, and entry^*a*^.

Mutant^a^	RNA replication	Assembly	Binding	Entry
Mutant 1	–	–	–	↓
Mutant 2	–	–	–	↓
Mutant 3	–	↓	–	↓
Mutant 4	–	↓	–	↓
Mutant 5	–	↓	–	↓
Mutant 6	–	↓	–	↑

*^*a*^Symbols are as follows: –, no detectable effects; ↑, increase; ↓, decrease.*

As E protein domain III plays an important role in mediating virus–cell interaction, we continued to evaluate the impact of mutations on the process of virus binding and entry. As expected, attenuating mutations did not simply block the infectivity of the virus through the process of virus assembly. The binding and entry activities of these attenuated variants had been destroyed. A previous study showed that loop III peptides (E-362-370) could prevent JEV binding to BHK-21 cells, and this finding is consistent with our results (Li et al., 2012). We also found the reason for the enhanced infectivity of mutant 6; that is, the mutation significantly enhanced the relative entry activity, even though its attachment capacity decreased slightly. In DENV2, an RGD motif (E-388-390 in TMUV) was crucial for the infection of mammalian cells but not C6/36 cells (Erb et al., 2010). The importance of the RGD motif for infecting mammalian cells and maintaining the thermal stability of virus particles was confirmed in YFV (van der Most et al., 1999). Moreover, the RGD motif has been previously shown to act as a recognition site for integrin receptors and to affect the entry process (Chu and Ng, 2004; Wang et al., 2020). The domain III lateral ridge containing this sequence is the recognition epitope of various antibodies, which could inhibit virus infection by inhibition of a postentry step (Zhao et al., 2020). These findings potentially indicate that in this motif, the epitope recognized by antibodies may have some potential connection with the sites that affect entry. Alanine mutations led to an increase in mutant 6 entry activity, perhaps affecting the interaction between the virus and receptors. However, in viruses without the RGD motif, the functionality of this sequence needs to be further studied. We performed homology modeling analysis of the mutants and further predicted their structure, and the findings revealed that the mutant 6 domain III lateral ridge was smoother than that in the WT virus, which may facilitate the entry process (Figure 6F).

Since the E protein participates in all aspects of the flavivirus life cycle, except genome replication, and shows structural specificity, it is widely used in drug development, antigen epitope screening, and vaccine development (Wu and Lin, 2001; Li et al., 2016; Cabral-Miranda et al., 2019). In fact, studies have shown that the mutations that appear during passage of the virus, or escape mutations obtained by antibody pressure screening, are mostly located in the functional domains (Serafin and Aaskov, 2001; Ramond et al., 2019). Our study revealed the important role of several residues in the loop regions of TMUV E protein domain III in viral proliferation. Our data highlight that E332, E365-367, and E388-390 are involved in TMUV virion assembly based on the subcellular fractionation and packaging system. The attenuation caused by the mutations (M304A.C305A.S306A, V312A, T332A, T332S, and S365A.S366A.T367A) was also attributed to the loss of relative entry activity (Table 3). Additionally, the S388A.G389A.K390A mutation increased viral infectivity *in vitro* by enhancing the relative entry activity, which may be because the mutation makes the structure more conducive to the entry process.

## Data Availability Statement

The original contributions presented in the study are included in the article/supplementary material, further inquiries can be directed to the corresponding author/s.

## Author Contributions

All authors listed have made a substantial, direct and intellectual contribution to the work, and approved it for publication.

## Conflict of Interest

The authors declare that the research was conducted in the absence of any commercial or financial relationships that could be construed as a potential conflict of interest.

## References

[B1] Allison S. L. Stiasny K. Stadler K. Mandl C. W. Heinz F. X. (1999). Mapping of functional elements in the stem-anchor region of tick-borne encephalitis virus envelope protein E. *J. Virol.* 73 5605–5612. 10.1128/jvi.73.7.5605-5612.1999 10364309 PMC112618

[B2] Cabral-Miranda G. Lim S. M. Mohsen M. O. Pobelov I. V. Roesti E. S. Heath M. D. (2019). *Zika Virus*-derived e-diii protein displayed on immunologically optimized VLPS induces neutralizing antibodies without causing enhancement of dengue virus infection. *Vaccines (Basel)* 7:72. 10.3390/vaccines7030072 31340594 PMC6789886

[B3] Chen S. He Y. Zhang R. Liu P. Yang C. Wu Z. (2018a). Establishment of a reverse genetics system for duck Tembusu virus to study virulence and screen antiviral genes. *Antiviral. Res.* 157 120–127. 10.1016/j.antiviral.2018.06.016 30057296

[B4] Chen S. Yang C. Zhang J. Wu Z. Wang M. Jia R. (2018b). Conserved active-site residues associated with OAS enzyme activity and ubiquitin-like domains are not required for the antiviral activity of goOASL protein against avian tembusu virus. *Viruses* 10:371. 10.3390/v10070371 30011971 PMC6071104

[B5] Chin J. F. Chu J. J. Ng M. L. (2007). The envelope glycoprotein domain III of dengue virus serotypes 1 and 2 inhibit virus entry. *Microb. Infect.* 9 1–6. 10.1016/j.micinf.2006.09.009 17196419

[B6] Chiou S. S. Fan Y. C. Crill W. D. Chang R. Y. Chang G. J. (2012). Mutation analysis of the cross-reactive epitopes of Japanese encephalitis virus envelope glycoprotein. *J. Gen. Virol.* 93(Pt 6) 1185–1192. 10.1099/vir.0.040238-0 22337639

[B7] Chu J. J. Ng M. L. (2004). Interaction of West Nile virus with alpha v beta 3 integrin mediates virus entry into cells. *J. Biol. Chem.* 279 54533–54541. 10.1074/jbc.m410208200 15475343

[B8] Cruz-Oliveira C. Freire J. M. Conceição T. M. Higa L. M. Castanho M. A. Da Poian A. T. (2015). Receptors and routes of dengue virus entry into the host cells. *FEMS Microbiol. Rev.* 39 155–170. 10.1093/femsre/fuu004 25725010

[B9] de Wispelaere M. Yang P. L. (2012). Mutagenesis of the DI/DIII linker in dengue virus envelope protein impairs viral particle assembly. *J. Virol.* 86 7072–7083. 10.1128/jvi.00224-12 22532681 PMC3416339

[B10] Du R. Wang M. Hu Z. Wang H. Deng F. (2015). An in vitro recombination-based reverse genetic system for rapid mutagenesis of structural genes of the Japanese encephalitis virus. *Virol. Sin.* 30 354–362. 10.1007/s12250-015-3623-2 26463213 PMC8200878

[B11] Erb S. M. Butrapet S. Moss K. J. Luy B. E. Childers T. Calvert A. E. (2010). Domain-III FG loop of the dengue virus type 2 envelope protein is important for infection of mammalian cells and *Aedes aegypti* mosquitoes. *Virology* 406 328–335. 10.1016/j.virol.2010.07.024 20708768

[B12] Fan J. Liu Y. Xie X. Zhang B. Yuan Z. (2013). Inhibition of Japanese encephalitis virus infection by flavivirus recombinant E protein domain III. *Virol. Sin.* 28 152–160. 10.1007/s12250-013-3331-8 23709058 PMC8208458

[B13] Fritz R. Blazevic J. Taucher C. Pangerl K. Heinz F. X. Stiasny K. (2011). The unique transmembrane hairpin of flavivirus fusion protein E is essential for membrane fusion. *J. Virol.* 85 4377–4385. 10.1128/jvi.02458-10 21325407 PMC3126228

[B14] Gehrke R. Ecker M. Aberle S. W. Allison S. L. Heinz F. X. Mandl C. W. (2003). Incorporation of tick-borne encephalitis virus replicons into virus-like particles by a packaging cell line. *J. Virol.* 77 8924–8933. 10.1128/jvi.77.16.8924-8933.2003 12885909 PMC167216

[B15] He Y. Liu P. Wang T. Wu Y. Lin X. Wang M. (2019). Genetically stable reporter virus, subgenomic replicon and packaging system of duck Tembusu virus based on a reverse genetics system. *Virology* 533 86–92. 10.1016/j.virol.2019.05.003 31136895

[B16] Kostyuchenko V. A. Lim E. X. Y. Zhang S. Fibriansah G. Ng N. S. Ooi J. S. G. (2016). Structure of the thermally stable Zika virus. *Nature* 533 425–428. 10.1038/nature17994 27093288

[B17] Lee E. Lobigs M. (2000). Substitutions at the putative receptor-binding site of an encephalitic flavivirus alter virulence and host cell tropism and reveal a role for glycosaminoglycans in entry. *J. Virol.* 74 8867–8875. 10.1128/jvi.74.19.8867-8875.2000 10982329 PMC102081

[B18] Lee E. Lobigs M. (2008). E protein domain III determinants of yellow fever virus 17D vaccine strain enhance binding to glycosaminoglycans, impede virus spread, and attenuate virulence. *J. Virol.* 82 6024–6033. 10.1128/jvi.02509-07 18400851 PMC2395160

[B19] Lee P. D. Mukherjee S. Edeling M. A. Dowd K. A. Austin S. K. Manhart C. J. (2130). The Fc region of an antibody impacts the neutralization of West Nile viruses in different maturation states. *J. Virol.* 87 13729–13740. 10.1128/jvi.02340-13 24109224 PMC3838241

[B20] Li C. Bai X. Meng R. Shaozhou W. Zhang Q. Hua R. (2016). Identification of a new broadly cross-reactive epitope within domain III of the duck tembusu virus E protein. *Sci. Rep.* 6:36288. 10.1038/srep36288 PMC5099753 27824100

[B21] Li C. Zhang L. Y. Sun M. X. Li P. P. Huang L. Wei J. C. (2012). Inhibition of Japanese encephalitis virus entry into the cells by the envelope glycoprotein domain III (EDIII) and the loop3 peptide derived from EDIII. *Antiviral. Res.* 94 179–183. 10.1016/j.antiviral.2012.03.002 22465300

[B22] Liang T. Liu X. Qu S. Lv J. Yang L. Zhang D. (2019). Pathogenicity of egg-type duck-origin isolate of Tembusu virus in Pekin ducklings. *BMC Vet. Res.* 15:362. 10.1186/s12917-019-2136-x 31651323 PMC6813075

[B23] Lin S. R. Zou G. Hsieh S. C. Qing M. Tsai W. Y. Shi P. Y. (2011). The helical domains of the stem region of dengue virus envelope protein are involved in both virus assembly and entry. *J. Virol.* 85 5159–5171. 10.1128/jvi.02099-10 21367896 PMC3126166

[B24] Liu H. Liu Y. Wang S. Zhang Y. Zu X. Zhou Z. (2015). Structure-based mutational analysis of several sites in the E protein: implications for understanding the entry mechanism of Japanese encephalitis virus. *J. Virol.* 89 5668–5686. 10.1128/jvi.00293-15 25762738 PMC4442514

[B25] Mandl C. W. Allison S. L. Holzmann H. Meixner T. Heinz F. X. (2000). Attenuation of tick-borne encephalitis virus by structure-based site-specific mutagenesis of a putative flavivirus receptor binding site. *J. Virol.* 74 9601–9609. 10.1128/jvi.74.20.9601-9609.2000 11000232 PMC112392

[B26] Matsui K. Gromowski G. D. Li L. Barrett A. D. (2010). Characterization of a dengue type-specific epitope on dengue 3 virus envelope protein domain III. *J. Gen. Virol.* 91(Pt 9) 2249–2253. 10.1099/vir.0.021220-0 20444995 PMC3052520

[B27] Mohd Ropidi M. I. Khazali A. S. Nor Rashid N. Yusof R. (2020). Endoplasmic reticulum: a focal point of Zika virus infection. *J. Biomed. Sci.* 27:27. 10.1186/s12929-020-0618-6 PMC6971992 31959174

[B28] Nickells J. Cannella M. Droll D. A. Liang Y. Wold W. S. Chambers T. J. (2008). Neuroadapted yellow fever virus strain 17D: a charged locus in domain III of the E protein governs heparin binding activity and neuroinvasiveness in the SCID mouse model. *J. Virol.* 82 12510–12519. 10.1128/jvi.00458-08 18842715 PMC2593324

[B29] Plante J. A. Torres M. Huang C. Y. Beasley D. W. C. (2016). Plasticity of a critical antigenic determinant in the West Nile virus NY99 envelope protein domain III. *Virology* 496 97–105. 10.1016/j.virol.2016.05.024 27284640 PMC4969113

[B30] Ramond P. Siano R. Schmitt S. de Vargas C. Marié L. Memery L. (2019). Deep mutational scanning comprehensively maps how zika envelope protein mutations affect viral growth and antibody escape. *J. Virol.* 93:e01291–19. 10.1128/JVI.01291-19 PMC6854493 31511387

[B31] Ryman K. D. Ledger T. N. Campbell G. A. Watowich S. J. Barrett A. D. (1998). Mutation in a 17D-204 vaccine substrain-specific envelope protein epitope alters the pathogenesis of yellow fever virus in mice. *Virology* 244 59–65. 10.1006/viro.1998.9057 9581778

[B32] Serafin I. L. Aaskov J. G. (2001). Identification of epitopes on the envelope (E) protein of dengue 2 and dengue 3 viruses using monoclonal antibodies. *Arch. Virol.* 146 2469–2479. 10.1007/s007050170017 11811694

[B33] Sun M. Zhang L. Cao Y. Wang J. Yu Z. Sun X. (2020). Basic amino acid substitution at residue 367 of the envelope protein of tembusu virus plays a critical role in pathogenesis. *J Virol* 94:e02011–19. 32024774 10.1128/JVI.02011-19 PMC7108846

[B34] Unni S. K. Rùžek D. Chhatbar C. Mishra R. Johri M. K. Singh S. K. (2011). Japanese encephalitis virus: from genome to infectome. *Microbes Infect.* 13 312–321. 10.1016/j.micinf.2011.01.002 21238600

[B35] van der Most R. G. Corver J. Strauss J. H. (1999). Mutagenesis of the RGD motif in the yellow fever virus 17D envelope protein. *Virology* 265 83–95. 10.1006/viro.1999.0026 10603320

[B36] Wang S. Zhang Q. Tiwari S. K. Lichinchi G. Yau E. H. Hui H. (2020). Integrin alphavbeta5 internalizes zika virus during neural stem cells infection and provides a promising target for antiviral therapy. *Cell Rep.* 30 969–983 e4. 31956073 10.1016/j.celrep.2019.11.020 PMC7293422

[B37] Watterson D. Kobe B. Young P. R. (2012). Residues in domain III of the dengue virus envelope glycoprotein involved in cell-surface glycosaminoglycan binding. *J. Gen. Virol.* 93(Pt 1) 72–82. 10.1099/vir.0.037317-0 21957126

[B38] Wu S. C. Lin C. W. (2001). Neutralizing peptide ligands selected from phage-displayed libraries mimic the conformational epitope on domain III of the Japanese encephalitis virus envelope protein. *Virus Res.* 76 59–69. 10.1016/s0168-1702(01)00246-5 11376846

[B39] Yan D. Shi Y. Wang H. Li G. Li X. Wang B. (2018). A single mutation at position 156 in the envelope protein of tembusu virus is responsible for virus tissue tropism and transmissibility in ducks. *J. Virol.* 92:e00427–18. 29899104 10.1128/JVI.00427-18 PMC6096821

[B40] Yang L. Liang T. Lv J. Qu S. Meng R. Yang B. (2021). Substantial attenuation of virulence of tembusu virus strain ps is determined by an arginine at residue 304 of the envelope protein. *J. Virol.* 95:e02331–20. 33328312 10.1128/JVI.02331-20 PMC8094961

[B41] Yu G. Lin Y. Tang Y. Diao Y. (2018). Evolution of tembusu virus in ducks, chickens, geese, sparrows, and mosquitoes in northern china. *Viruses* 10:485. 10.3390/v10090485 30201873 PMC6164154

[B42] Yu I. M. Zhang W. Holdaway H. A. Li L. Kostyuchenko V. A. Chipman P. R. (2008). Structure of the immature dengue virus at low pH primes proteolytic maturation. *Science* 319 1834–1837. 10.1126/science.1153264 18369148

[B43] Zhang X. Jia R. Shen H. Wang M. Yin Z. Cheng A. (2017). Structures and functions of the envelope glycoprotein in flavivirus infections. *Viruses* 9:338. 10.3390/v9110338 29137162 PMC5707545

[B44] Zhao H. Xu L. Bombardi R. Nargi R. Deng Z. Errico J. M. (2020). Mechanism of differential Zika and dengue virus neutralization by a public antibody lineage targeting the DIII lateral ridge. *J. Exp. Med.* 217:e20191792. 10.1084/jem.20191792 PMC7041715 31757867

